# Completely extraperitoneal approach via midline incision through the pre-transversalis fascia space for resection of retroperitoneal tumor: a case report

**DOI:** 10.1093/jscr/rjag1028

**Published:** 2026-11-10

**Authors:** Jiaxin Wu, Shaoxiong Yi, Guizeng Chen, Jinshi Chen, Jianlong Jiang, Dongjie Yang

**Affiliations:** Digestive Medicine Center, The Seventh Affiliated Hospital, Sun Yat-sen University, No. 628, Zhenyuan Road, Shenzhen 518107, China; Digestive Medicine Center, The Seventh Affiliated Hospital, Sun Yat-sen University, No. 628, Zhenyuan Road, Shenzhen 518107, China; Digestive Medicine Center, The Seventh Affiliated Hospital, Sun Yat-sen University, No. 628, Zhenyuan Road, Shenzhen 518107, China; Digestive Medicine Center, The Seventh Affiliated Hospital, Sun Yat-sen University, No. 628, Zhenyuan Road, Shenzhen 518107, China; Digestive Medicine Center, The Seventh Affiliated Hospital, Sun Yat-sen University, No. 628, Zhenyuan Road, Shenzhen 518107, China; Digestive Medicine Center, The Seventh Affiliated Hospital, Sun Yat-sen University, No. 628, Zhenyuan Road, Shenzhen 518107, China

**Keywords:** retroperitoneal tumor, totally extraperitoneal approach, surgery

## Abstract

Surgical resection is the mainstay of treatment for retroperitoneal tumors. Conventional surgical approaches include the transperitoneal approach and the extraperitoneal approach via the lateral abdominal wall. We report a 36-year-old male with a left retroperitoneal tumor who underwent tumor resection via a midline incision through the pre-transversalis fascia space using a completely extraperitoneal approach along. Compared with the aforementioned approaches, this new technique avoids division of the abdominal wall musculature while preserving the integrity of the peritoneal cavity. The patient was discharged uneventfully on postoperative Day 3. Histopathological examination revealed Castleman disease. Follow-up computed tomography 3 months postoperatively showed no evidence of tumor recurrence. This approach has not been previously described in the literature. This case suggests that for tumors confined to the unilateral retroperitoneal space, it may serve as a safe and viable surgical alternative.

## Introduction

Primary retroperitoneal tumors are benign or malignant neoplasms originating in the retroperitoneal space, and surgical resection remains their principal treatment. Conventional surgical procedures include transperitoneal and lateral abdominal wall extraperitoneal approach. To our knowledge, a fully extraperitoneal approach via a midline incision through the pre-transversalis fascia space has not been previously reported in the literature. Compared with conventional approaches, this technique preserves the integrity of both the peritoneal cavity and the abdominal wall musculature and may facilitate postoperative recovery. Recently, we successfully performed this procedure in a patient with a retroperitoneal tumor. The clinical details of this case and a review of the relevant literature are presented below.

## Case report

A 36-year-old male patient was admitted to our hospital with a left retroperitoneal mass, which had been identified by computed tomography (CT) scan at an outside hospital after the patient experienced a one-month history of distending pain in the left flank and abdomen. The patient had no significant past medical history. Contrast-enhanced CT of the chest, abdomen, and pelvis showed a 5 × 6 cm hypervascular paravertebral mass at the upper level of the left psoas major muscle. A neurogenic tumor was suspected (Fig. 1).

**Figure 1:**
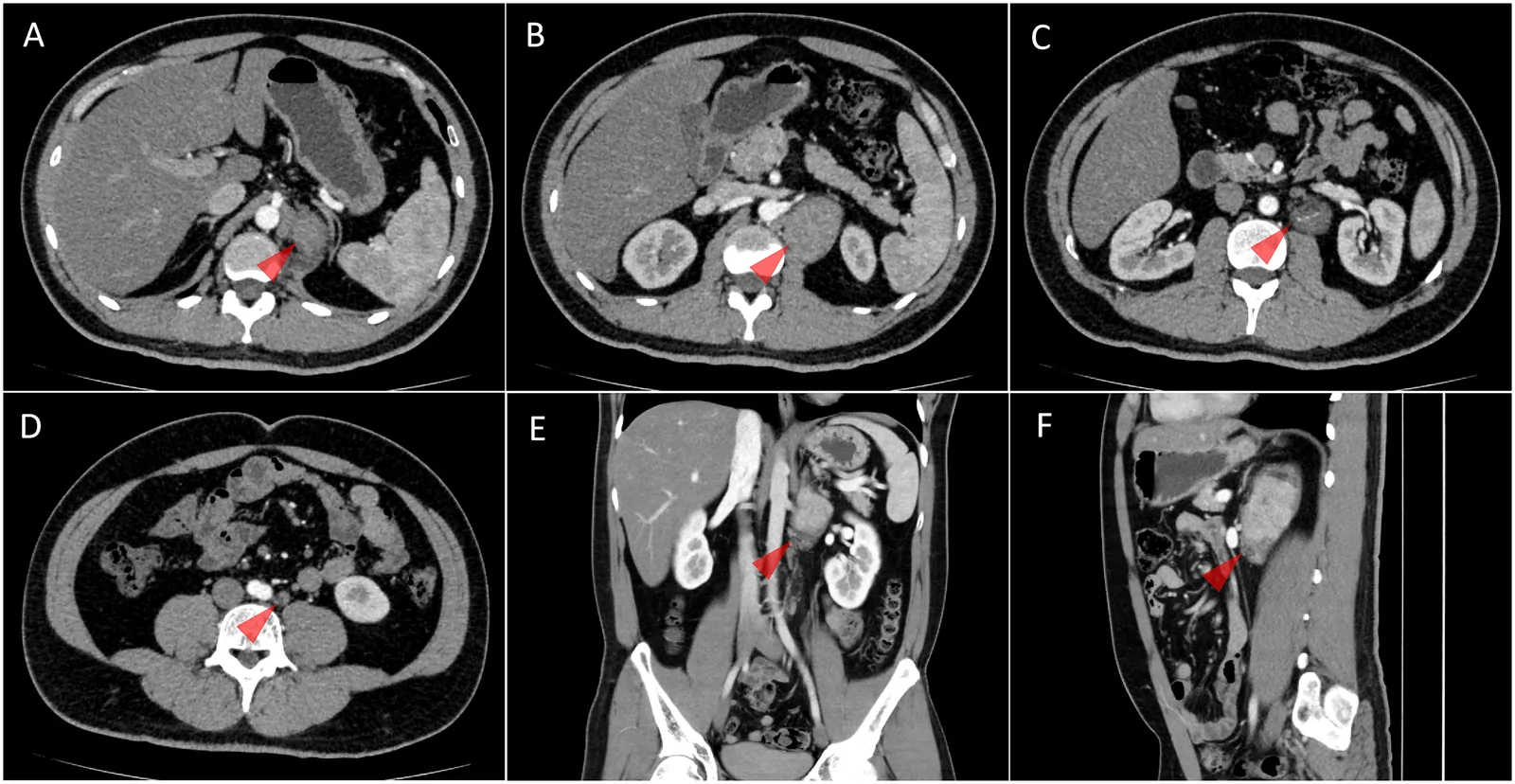
Preoperative contrast-enhanced CT showing a main tumor measuring ~5 × 6 cm. (A) Abdominal aortic level. (B) Renal artery level. (C) Renal hilum level. (D) Enlarged lymph nodes at the aortic bifurcation. (E) Coronal image at the abdominal aortic level. (F) Sagittal image at the psoas major level. Red arrows indicate the tumor or enlarged lymph nodes.

After multidisciplinary team discussion, the patient underwent complete extraperitoneal resection of the left retroperitoneal tumor via a midline incision through the pre-transversalis fascia space (Fig. 2). The principal operative steps were as follows. First, with the patient in the supine position, a midline incision was made from the xiphoid process to 3 cm below the umbilicus (Fig. 3A). Second, the layers of the abdominal wall were incised sequentially down to the superficial surface of the transversalis fascia, thereby entering the pre-transversalis fascia space. Dissection was carried leftward along the superficial surface of the transversalis fascia (i.e. the deep surface of the fascia of the rectus abdominis and transversus abdominis), and then continued along the anterior surface of the quadratus lumborum and psoas major (i.e. the posterior surface of the posterior renal fascia) to the left margin of the abdominal aorta (Fig. 3B and C). Third, the anterior aspect of the tumor was dissected, followed by its medial aspect, and the left lumbar arteries and veins entering the tumor were ligated and divided. The main tumor mass, together with the enlarged caudal lymph nodes was completely resected (Fig. 3D). Fourth, abdominal wall retraction was released, and the dissected retroperitoneal space spontaneously return to its anatomical position (Fig. 3E). Finally, the surgical specimen was opened for pathological examination. Its capsule was intact.

**Figure 2:**
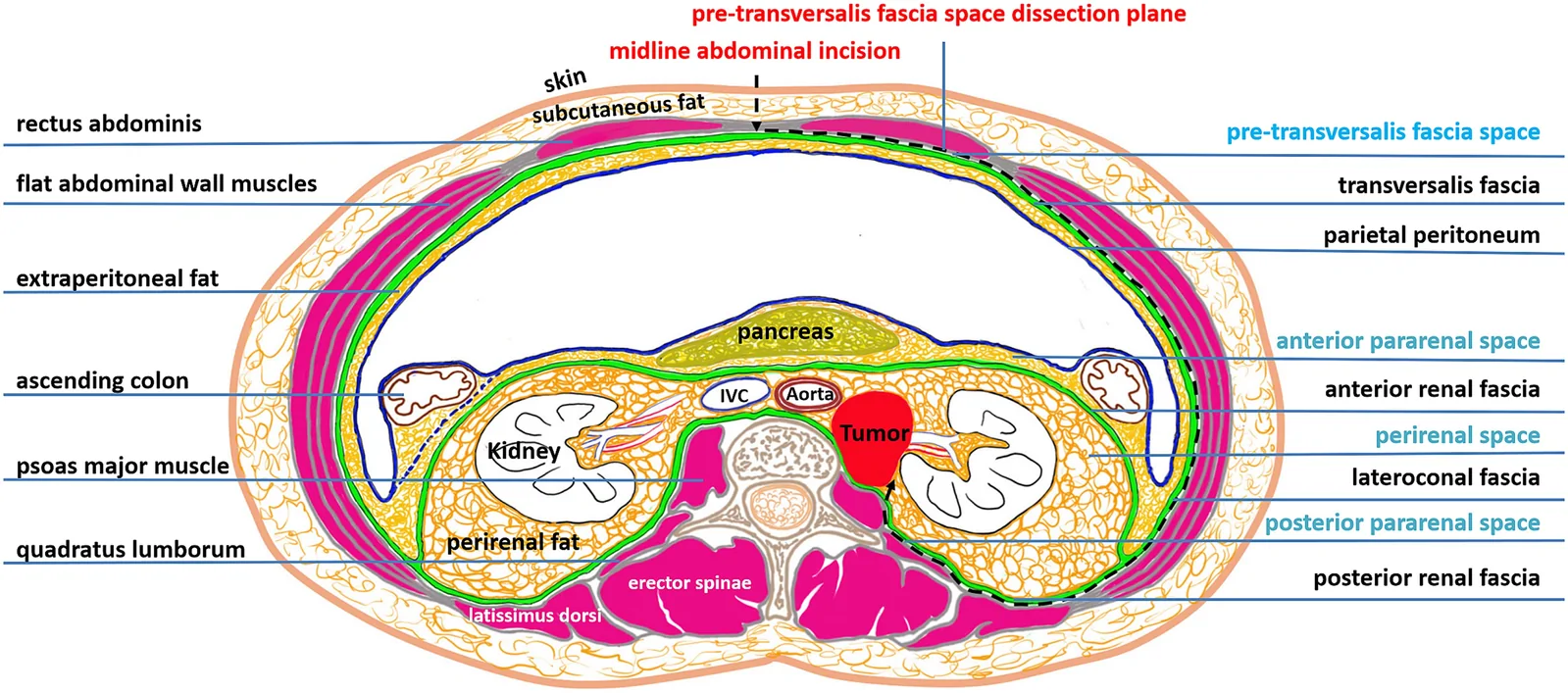
Fasciae and interfascial spaces of the abdominal wall, and surgical incisions and planes of dissection. The transversalis fascia(indicated by the green line) lines the inner surface of the rectus abdominis and the flat abdominal wall muscles, and the pre-transversalis fascia space located between the fascia and these muscles. Dorsally, the transversalis fascia continuous as the lateroconal fascia, which divides into the anterior and posterior renal fasciae. The posterior renal fascia lies along the surface of the psoas major and quadratus lumborum, with the posterior pararenal space situated between them. Accordingly, the pre-transversalis fascia space therefore communicates with the posterior pararenal space. The dashed line shows the surgical route: a midline incision is made to the surface of the transversalis fascia, through which the pre-transversalis fascia space is entered. Dissection then continues into the posterior pararenal space, followed by tumor resected.

**Figure 3:**
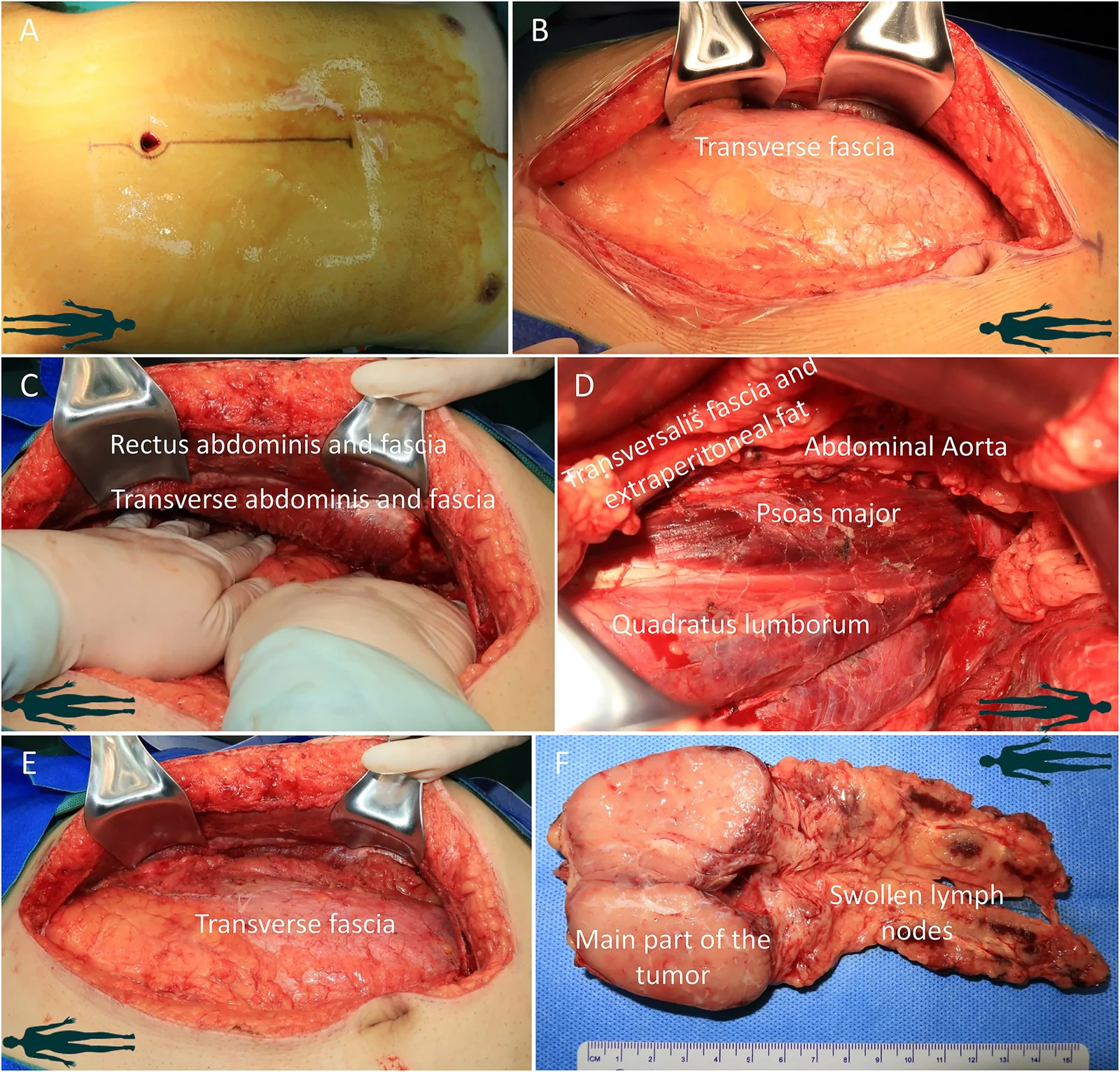
Operative procedure. (A) Upper midline abdominal incision, ~20 cm long. (B) After division of the linea alba, dissection proceeded along the superficial surface of the transversalis fascia. (C) At the posterior abdominal wall, dissection proceeded along the superficial surface of the psoas fascia. (D) Operative field after tumor resection. (E) After release of retraction, the retroperitoneal space returned spontaneously to its anatomical position. (F) Cut surface of the specimen.

No peritoneal injury occurred during dissection, and the peritoneal cavity remained intact. Operative time was 220 min, and estimated blood loss was 20 ml. The patient was discharged on postoperative Day 3. Histopathological examination showed an encapsulated lesion with prominent lymphoid hyperplasia and follicular architecture. In some areas, the mantle-zone lymphocytes formed a hyaline, concentric onion-skin pattern, accompanied by marked vascular proliferation. Numerous plasma cells and proliferating vessels were present in the interfollicular regions. Immunohistochemistry showed ‘positive expression of CD20, CD3, and CD23’. The diagnosis was hyaline vascular-type Castleman disease (Fig. 4).

**Figure 4:**
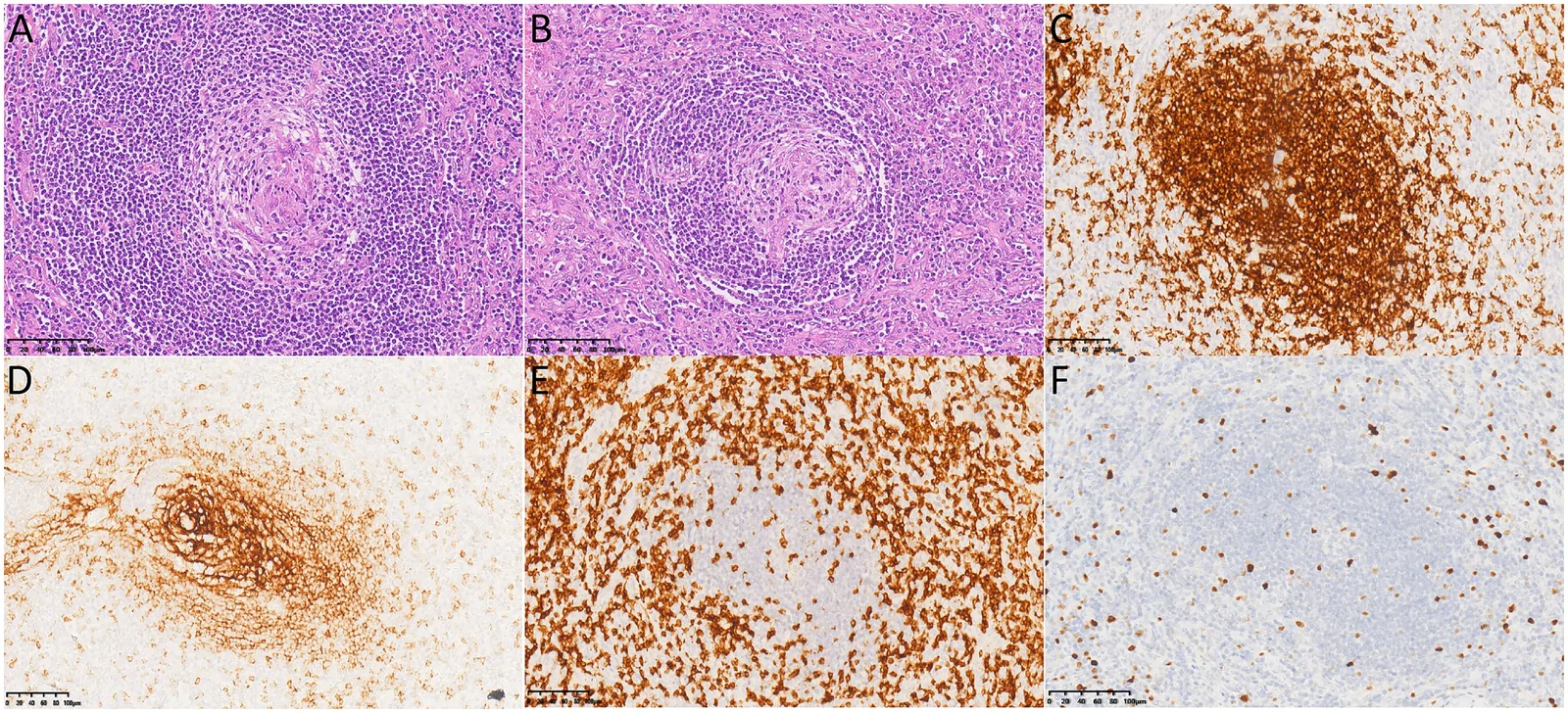
Histopathological and immunohistochemical features of the lymph node lesion, consistent with hyaline vascular-type Castleman disease. (A) An atrophic germinal center is surrounded by concentric layers of mantle-zone lymphocytes, producing the characteristic onion-skin appearance (H&E, ×200). (B) A hyalinized small vessel penetrates the germinal center, creating a lollipop appearance (H&E, ×200). (C) CD20 staining shows mantle-zone B lymphocytes arranged concentrically in an onion-skin pattern (×200). (D) CD23 staining shows marked proliferation and disruption of the follicular dendritic cell network (×200). (E) CD3 staining shows proliferation of interfollicular T lymphocytes (×200). (F) Ki-67 staining shows a low proliferative index (×200).

At outpatient follow-up 3 months postoperatively, contrast-enhanced CT showed no tumor recurrence, and the local anatomy appeared normal (Fig. 5).

**Figure 5:**
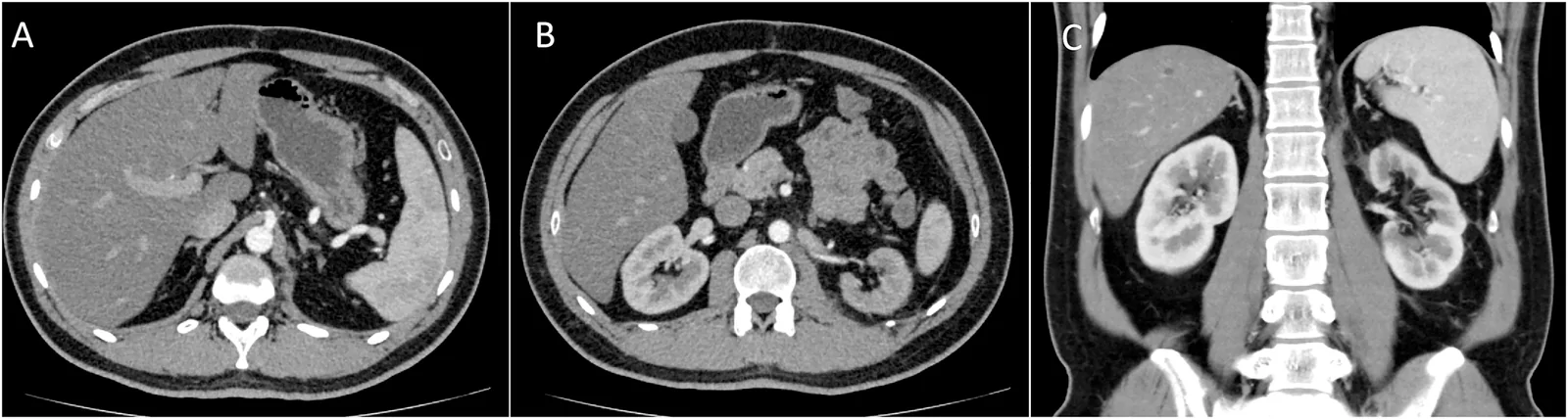
Contrast-enhanced CT 3 months after surgery. (A) Abdominal aortic level. (B) Renal artery level. (C) Coronal image at the psoas major level.

## Discussion

Primary retroperitoneal tumors encompass benign and malignant neoplasms of varied histological origin [1–5]. Castleman disease is a rare lymphoproliferative disorder classified clinically as unicentric (UCD) or multicentric subtypes [6, 7, 9]. The present patient was diagnosed with UCD. Because its imaging features overlap with those of other hypervascular retroperitoneal tumors, definitive diagnosis usually depends on histopathological examination and immunohistochemical analysis [5, 8, 9]. Surgical resection remains the primary treatment modality for retroperitoneal tumors, including UCD [1–3].

Conventional surgical procedures for retroperitoneal tumors include transperitoneal or lateral extraperitoneal approach [3, 10–13]. Transperitoneal approach provides broad exposure and remains appropriate for very large tumors, marked visceral displacement, combined organ resection, or major vascular reconstruction [3, 11–13]. However, it requires entry into the peritoneal cavity and manipulation of intra-abdominal organs. The lateral extraperitoneal approach avoids peritoneal entry but may provide limited exposure and disrupt abdominal wall muscles or nerves [10–12]. The totally extraperitoneal tumor resection via midline incision through the pre-transversalis fascia space performed in this patient avoided these drawbacks.

Safe application of this approach requires detailed understanding of retroperitoneal spaces and fasciae anatomy [14, 15]. Currently, the anatomy of the abdominal wall fasciae and their associated spaces, particularly the continuity between them, remains controversial. Based on the literature and our clinical experience, we believe that the anterior and posterior renal fasciae divide the retroperitoneum into anterior pararenal, perirenal, and posterior pararenal spaces and are continuous with fascial planes of the lateral and anterior abdominal walls [14, 15]. The psoas fascia is continuous with the deep fasciae of the transversus abdominis and rectus abdominis, while the renal fasciae continue laterally with the lateroconal and transversalis fasciae. Dissection along the superficial surface of the transversalis fascia therefore provides access toward the posterior pararenal plane while leaving the transversalis fascia, extraperitoneal fat, and parietal peritoneum between the operative field and peritoneal cavity. This layered barrier may help preserve peritoneal integrity and facilitate restoration of normal anatomy after retraction is released.

In summary, based on the surgical experience gained in the present case together with a review of the literature, the extraperitoneal approach via a midline abdominal incision with dissection in the pre-transversalis fascial plane may serve as a safe and viable surgical option for retroperitoneal tumors that are confined to the unilateral retroperitoneal space without involvement of the abdominal viscera or the retroperitoneal major vessels.

## References

[1] Sun S, Cardona K, Tseng WW. Surgical management of retroperitoneal liposarcoma: opportunities for multimodality treatment, including systemic therapy. Cancer Med 2025;14:e71129. 10.1002/cam4.71129 PMC12332537 40778536

[2] Caturegli I, Lilley EJ, Fairweather M et al. Retroperitoneal sarcoma (RPS): a systematic review. Curr Oncol Rep 2025;27:856–70. 10.1007/s11912-025-01690-2 40465137

[3] Bonvalot S, Raut CP, Pollock RE et al. Technical considerations in surgery for retroperitoneal sarcomas: position paper from E-Surge, a master class in sarcoma surgery, and EORTC-STBSG. Ann Surg Oncol 2012;19:2981–91. 10.1245/s10434-012-2342-2 22476756

[4] Mack T, Purgina B. Updates in pathology for retroperitoneal soft tissue sarcoma. Curr Oncol 2022;29:6400–18. 10.3390/curroncol29090504 PMC9497884 36135073

[5] Messiou C, Morosi C. Imaging in retroperitoneal soft tissue sarcoma. J Surg Oncol 2018;117:25–32. 10.1002/jso.24891 PMC5836919 29193092

[6] Fajgenbaum DC, Uldrick TS, Bagg A et al. International, evidence-based consensus diagnostic criteria for HHV-8-negative/idiopathic multicentric Castleman disease. Blood 2017;129:1646–57. 10.1182/blood-2016-10-746933 PMC5364342 28087540

[7] Wu D, Lim MS, Jaffe ES. Pathology of Castleman disease. Hematol Oncol Clin North Am 2018;32:37–52. 10.1016/j.hoc.2017.09.004 29157618

[8] Bonekamp D, Horton KM, Hruban RH et al. Castleman disease: the great mimic. RadioGraphics 2011;31:1793–807. 10.1148/rg.316115502 21997995

[9] Talat N, Belgaumkar AP, Schulte K-M. Surgery in Castleman’s disease. Ann Surg 2012;255:677–84. 10.1097/SLA.0b013e318249dcdc 22367441

[10] Sato Y, Matsuhashi N, Sugie Y et al. Total laparoscopic resection by medial-retroperitoneal approach using virtual navigation: two case reports of primary retroperitoneal schwannoma. World J Surg Oncol 2022;20:3. 10.1186/s12957-021-02483-0 PMC8725496 34980150

[11] Fairweather M, Gonzalez RJ, Strauss D et al. Current principles of surgery for retroperitoneal sarcomas. J Surg Oncol 2018;117:33–41. 10.1002/jso.24919 29315649

[12] Hou Z, Xie Q, Qiu G et al. Safety and efficacy of laparoscopic transperitoneal versus retroperitoneal resection for benign retroperitoneal tumors: a retrospective cohort study. Surg Endosc 2023;37:9299–309. 10.1007/s00464-023-10504-0 37884734

[13] Gronchi A, Pollock R. Surgery in retroperitoneal soft tissue sarcoma: a call for a consensus between Europe and North America. Ann Surg Oncol 2011;18:2107–10. 10.1245/s10434-011-1746-8 21537862

[14] Coffin A, Boulay-Coletta I, Sebbag-Sfez D et al. Radioanatomy of the retroperitoneal space. Diagn Interv Imaging 2015;96:171–86. 10.1016/j.diii.2014.06.015 25547251

[15] Lee SL, Ku YM, Rha SE. Comprehensive reviews of the interfascial plane of the retroperitoneum: normal anatomy and pathologic entities. Emerg Radiol 2010;17:3–11. 10.1007/s10140-009-0809-7 19399541

